# Sustaining Health Promotion in Australian Supermarkets: Qualitative Perspectives From the Reach for the Stars Programme

**DOI:** 10.1002/hpja.70228

**Published:** 2026-08-11

**Authors:** Rebecca Bennett, Laura Duff, Kate Mallia, Steven Allender, Bettina Backman, Miranda Blake, Shaan Naughton, Carmen Vargas

**Affiliations:** ^1^ Institute for Health Transformation, Global Centre for Preventive Health and Nutrition Deakin University Geelong Australia; ^2^ Latrobe Community Health Service Morwell Australia; ^3^ Latrobe Health Assembly Morwell Australia

**Keywords:** food environment, healthy food retail, programme sustainability, qualitative, supermarket intervention

## Abstract

**Issue Addressed:**

Supermarkets influence dietary behaviours, but sustaining health‐promoting interventions in these settings is challenging. This study explored factors shaping the long‐term sustainability of *Reach for the Stars*, a multi‐component intervention promoting products rated four or five health stars in independent supermarkets in the Latrobe Valley, regional Victoria, Australia.

**Methods:**

Semi‐structured interviews were conducted with health promotion staff, community members and supermarket owners and managers. Interviews were analysed using reflexive thematic analysis underpinned by a Big Q, constructionist approach, prioritising participants' experiences and perspectives rather than quantification.

**Results:**

Participants identified multiple factors influencing sustainability. Community engagement guided intervention design, ensuring materials met local needs, while trusting relationships between health promotion staff and retailers facilitated collaboration. Key challenges included limited ongoing funding, time constraints and inflexible organisational structures. Partnerships, adaptable resources and visible benefits to the community were highlighted as critical enablers. Participants also emphasised the importance of organisational support and leadership in sustaining programme practices over time.

**Conclusions:**

Sustaining supermarket‐based health promotion initiatives requires attention to relational, organisational and contextual factors beyond programme design. Active relationship‐building, collaborative partnerships and responsiveness to local context supported programme viability and retailer engagement.

**So What?:**

These findings provide practical insights for public health practitioners seeking to implement or maintain supermarket health initiatives. Emphasising community connection, partnership development and organisational support can strengthen the longevity and effectiveness of interventions aimed at promoting healthier food choices in retail settings.

## 1 Background

Unhealthy diets (i.e., those characterised by excessive intake of foods high in sugar, salt and saturated and low intake of fruits and vegetables) [1] have been recognised as a significant driver of the increase in preventable non‐communicable diseases globally [2]. This trend is reflected in Australia, where most people do not meet the national dietary guidelines [3]. The food retail environment (the places where people buy their food and drinks) can play a significant role in shaping population diets and health [4, 5, 6, 7, 8], however the characteristics of food retail environments can vary between contexts. For example, in rural and remote areas, residents often report poorer dietary intake and experience worse chronic disease profiles than their metropolitan counterparts [9, 10]. The Australian population living outside metropolitan areas experiences limited access to healthy food [11, 12] because of diverse and unique geographic location challenges [10].

In Australia, supermarkets play a key role in the food retail environment, as 63% of all calories purchased in Australia are purchased from supermarkets [6]. The Australian supermarket sector is also highly concentrated, with two major supermarket chains accounting for the majority of grocery sales nationally [13]. This market concentration gives supermarkets considerable influence over population diets through decisions regarding product availability, pricing, placement and promotion. Recent scrutiny of supermarket pricing and promotional practices has highlighted consumer concerns regarding the influence of supermarket commercial priorities on food retail environments [14, 15]. Previous studies have shown the relationship between purchasing behaviours and dietary behaviours [16], with less healthy food purchases leading to poorer dietary and health outcomes [17]. Despite Australian supermarkets offering fresh fruits, vegetables, meats and breads, recent studies show they often also extensively promote unhealthy foods and drinks, with greater discounts applied to unhealthy items [18]. Additionally, Australian supermarkets also place unhealthy items in key locations within the store (e.g., end‐of‐aisle displays) to further increase sales [19]. Previous studies have shown that consumers are strongly influenced by in‐store promotions when making purchasing decisions, including discounts, product placements and loyalty rewards [20, 21]. These marketing strategies can heavily influence consumer choices, often steering purchasing behaviours towards unhealthy options and contributing to broader public health concerns.

While much of the existing literature focuses on major supermarket chains [22, 23, 24], less attention has been paid to opportunities for promoting healthier food choices within independent supermarkets, particularly in regional communities experiencing socioeconomic disadvantage. However, long‐term sustainment of health‐promoting interventions in food retail settings can be difficult, as it is currently not well understood how to ensure a programme continues in the absence of continued external funding or support [25]. Despite often promising initial results, retail‐based interventions may not be sustained for various reasons [26], including competing commercial priorities of retailers, a lack of ongoing funding, limited staff engagement and challenges in adapting interventions to changing market conditions [27]. Particularly, the dynamic and competitive nature of the food retail sector often shifts focus towards profitability and customer preferences, making it challenging to prioritise and maintain public health‐focused initiatives over time [28]. This study aimed to examine the perspectives and experiences of key interest‐holders, including the barriers and enablers to sustaining a health‐promoting supermarket programme, to generate insights that can inform future public health initiatives in supermarket settings.

## 2 Methods

### 2.1 Study Design

The aim of this study was to explore barriers and enablers to sustaining the RFTS supermarket intervention from the perspectives of community members, retailers and health promotion staff [29]. RFTS is a Latrobe Health Innovation Zone initiative, funded by Latrobe Health Assembly and the Victorian Government.

### 2.2 Context

The Latrobe Valley, located in Gippsland, Eastern Victoria, Australia is a historically coal‐dependent region undergoing significant social, economic and health transitions in the context of industrial change and energy transition [30]. The Latrobe Health Assembly is a community‐driven governance body established in 2016 to foster collaboration between residents, service providers and policymakers in order to improve health and wellbeing outcomes in the Latrobe Valley [31]. The Latrobe Valley local government area faces high levels of socioeconomic disadvantage, as reflected in low scores on the Australian Bureau of Statistics Index of Relative Socio‐Economic Disadvantage (IRSD), as well as lower levels of education and skilled employment, indicated by the Index of Education and Occupation (IEO) [32].

RFTS is a supermarket‐based, healthy food marketing initiative managed by the Latrobe Community Health Service and the Latrobe Health Assembly, that was co‐designed by participating supermarkets and the wider community through a series of focus groups [33]. RFTS aims to make it easier for customers to shop, cook and eat healthier foods and drinks by improving the healthiness of local, independent supermarkets. The programme was adapted from a previous supermarket‐based intervention in another regional Victorian centre, the Eat Well@IGA programme in the City of Greater Bendigo [34]. RFTS was piloted in three supermarkets in 2022 and expanded to five total supermarkets during a second phase, from 2023 to 2025. RFTS utilises the Health Star Rating (HSR) front‐of‐pack nutrition labelling system [35] to identify and promote healthier food and drink choices within participating supermarkets. Intervention activities included messaging (i.e., ‘All fresh fruit and vegetables are 5 stars!’), promotional materials (i.e., Health Star Rating shelf tags on products 4 stars and above, and healthy recipe cards), a communications plan/social marketing campaign and staff training to build the capacity of supermarket staff.

#### 2.2.1 The Health Star Rating System

The HSR system was adopted nationally in 2014, as a voluntary measure. The system aimed to improve the healthiness of food and drinks purchased in Australia by guiding consumers towards healthier choices using a front‐of‐pack labelling system where foods were labelled from 0.5 (least healthy) to 5 stars (most healthy) [36]. However, the system has attracted criticism for its low uptake by food manufacturers, especially among unhealthy products [13] and a misalignment between the healthiness of products and the number of stars they receive due to the calculation algorithm [36]. Additionally, qualitative research with Australian consumers has highlighted concerns regarding the transparency of the calculation criteria, with consumers reporting that they had ‘little confidence’ in the system [37]. Despite these limitations, the HSR system was selected for RFTS because it was the nationally endorsed front‐of‐pack nutrition labelling system at the time of intervention implementation. To support healthier food choices, RFTS focused on promoting products receiving 4 or 5 Health Stars, alongside fresh fruit and vegetables, which are assigned a default rating of 5 Health Stars despite not routinely displaying the label.

### 2.3 Sampling and Recruitment

We used purposive sampling to identify participants who were involved in at least one programme stage (planning, implementing, and/or data collection). This included retail staff, retail store owners, community members and health promotion staff working for Latrobe Community Health Service and Latrobe Health Assembly. The RFTS project manager directly contacted 25 potential participants and invited them to participate in a one‐on‐one or group interview via e‐mail correspondence detailing the research aims and objectives. If participants agreed to participate, a plain language statement and consent form were provided, and an interview took place using Zoom [38] or over the phone.

### 2.4 Data Collection

Semi‐structured interviews were conducted by members of the research team (R.B. and/or C.V.) using an interview guide. The interview guide was developed inductively, based on the study aims, to elicit open‐ended narratives and allow participants to identify and elaborate on issues they considered important. The interview guide is available in File S1. This approach prioritised depth, context and the co‐construction of meaning between participants and researchers, rather than the measurement or quantification of views. The interview guide was iteratively reviewed, pilot tested and edited by the research team prior to recruitment. While the interview guide provided broad areas of inquiry, the interviews were responsive, allowing participants' perspectives to guide the flow of discussion. Not every question in the interview guide was asked in every interview; instead, topics were introduced flexibly and adapted according to participants' responses and the natural flow of conversation.

All interviews were conducted remotely, in English, and audio recorded between December 2024 and March 2025. Interview transcripts were either created by the Zoom software platform and checked for accuracy, or manually by R.B. in the case of phone interviews.

### 2.5 Analysis

The interview transcripts were coded and analysed thematically using QSR NVivo software [39]. This study adopted a Big Q qualitative approach [40], grounded in a constructivist paradigm, which prioritises depth, reflexivity and interpretive richness over completeness. In a Big Q qualitative approach, the pursuit of theoretical sufficiency replaces the traditional goal of data saturation, reflecting a shift towards a more flexible and context sensitive approach to analysis [40]. Rather than attempting to exhaust all possible themes or reach a point where no new insights were gained, researchers aimed to gather enough data to support and refine theoretical constructs meaningfully [40]. By embracing theoretical sufficiency, researchers acknowledge the inherently partial and situated nature of qualitative inquiry, allowing for a more pragmatic engagement within the epistemological foundations of Big Q. This study is also reported in accordance with the Standards for Reporting Qualitative Research (SRQR) guidelines [41], and a completed SRQR checklist is provided in File S2.

Coding was conducted inductively by a single author (R.B.), with no predefined codebook or inter‐coder reliability checks. This allowed for deep immersion in the data and recognition of the researcher's interpretive role in constructing meaning. This process involved R.B. rereading the transcripts to familiarise herself with the data, then grouping similar responses together to understand the main themes and perspectives.

As part of the reflexive thematic analysis process, R.B. developed visual mind maps to explore relationships between codes and emerging concepts across the dataset. These diagrams were used as an analytic tool to support interpretation and theme development by documenting early reflections on connections between ideas, participant experiences and potential thematic patterns. Key quotes from participants were used to illustrate findings.

### 2.6 Research Team and Reflexivity

The research team comprised university researchers affiliated with NHMRC‐funded Centre of Research Excellence in Food Retail Environments for Health (RE‐FRESH: Next Generation) and health promotion practitioners involved in implementing RFTS. Team members brought expertise in qualitative research, public health, business, programme implementation and sustainability. Two listed authors (L.D. and K.M.), who were interviewed as participants, provided contextual insight into the development and delivery of RFTS. Their dual roles were recognised as shaping both the production of data and its interpretation.

Data collection and primary analysis were led by university researchers who had no prior involvement in RFTS. This separation was intended to support independent interpretation of participant accounts while still allowing implementation staff to contribute contextual knowledge during manuscript development. Rather than seeking to eliminate researcher influence, we approached differing perspectives within the research team as analytically valuable.

As public health researchers committed to improving food environments, we were attentive to relational and organisational enablers of sustainability. We were particularly attentive to factors that supported or hindered the sustainability of health‐promoting interventions and acknowledge that alternative interpretations may have emphasised different aspects of participants' experiences.

To enhance trustworthiness and rigour, reflexive discussions were held throughout analysis, findings were interpreted by the research team and perspectives were sought from multiple interest‐holder groups (community members, retailers and health promotion staff) to allow triangulation of experiences.

### 2.7 Ethics Information

Ethics approval was obtained from Deakin University's Human Research Ethics Committee (2024/HE000289). All participants provided informed consent to participate. All but one participant consented to audio recording and the use of non‐identifiable quotes; for the interview conducted without recording, detailed notes were taken by R.B. with the participant's consent.

## 3 Results

### 3.1 Overview of Participants

Twelve participants (*n* = 8 women, 66%) were involved across two focus groups (with three and four participants each), and five one‐on‐one interviews. The two focus groups included members of the community (*n* = 4) who were part of the project advisory group at Latrobe Health Assembly, as well as health promotion staff from Latrobe Community Health Service and Latrobe Health Assembly. One‐on‐one interviews were conducted with retail store owners and managers at the participating stores (*n* = 5), and the main project officers from Latrobe Community Health Service (*n* = 3). The interviews were between 11 and 55 min, with an average length of 28 min.

Thematic analysis constructed three themes to describe how participants experienced RFTS: ‘Listening to community and retailer voices improved programme fit’, ‘Trusting relationships enabled implementation and change’ and ‘Scaling up required organisational commitment and investment’. A list of all codes is available in File S3.

### 3.2 Listening to Community and Retailer Voices Improved Programme Fit

Participants consistently described a need for a healthy food retail programme like RFTS in the Latrobe Valley. They explained that the community had a limited understanding of what foods and drinks were and were not healthy, and few ate according to recommendations from the Australian Dietary Guidelines, especially in terms of fruit and vegetable consumption. A community member, who is also a local teacher, commented:

[At] another school with very low socioeconomic [status], and the kids are just, you know, they just [eating unhealthy food] … it's also affecting their overall performance at school. They can't learn on this crap food, and they can't behave on this crap food. (Community member #1)

To address this need, participants explained how the co‐design process allowed the community to participate in designing a highly relevant and practical programme that would help them to improve their diets. As one community member explained:

Back when it first started, [they] did a series of focus groups with community … And they came up with some really, really strong key messages that helped to develop the program. (Community member #2)

From this consultation, a simple‐to‐follow intervention was designed. Participants discussed how the community wanted healthy, easy‐to‐prepare recipes, which guided the development of recipe cards that could be placed alongside other promotional materials in participating stores. Ultimately, the intervention was aiming to improve the healthiness of food purchasing

Improving the health literacy of the parents that you know like, if we could somehow influence that in the supermarket would be really powerful. So that would be something that I'd be interested to pursue, because at the end of the day the parents are buying the food. (Community member #3)

Informed by community experiences, the intervention was also designed to use positive messaging about healthy foods, instead of negative messaging about unhealthy foods, which was a concern for community mental health.

Supermarkets and retailers were identified as potential community leaders who could work to improve the health of the Latrobe Valley community. Health promotion staff discussed how they knew that supermarkets and retailers could play a key role and wanted to work together to achieve better community dietary health, as one staff member explained:

Supermarkets really are interested in working in these types of projects. They're interested because they're community members themselves. (Health promotion staff #1)

To work effectively with retailers, health promotion staff expressed the importance of listening to their feedback. Participants explained how they wanted retailers to ‘take ownership’ of the project and feel involved in the process. This included health promotion staff empowering retailer staff through training, which one retailer explained helped staff to feel more confident to explain the benefits of RFTS to customers:

[Staff] started appreciating [RFTS] a bit more, asking, then they felt confident, they wanted to know more about it. How it works. How can we have more information which we can feel more confident to pass on. It was really great for us. (Retailer #1)

Additionally, retailers explained how their own expertise and knowledge of their customer base helped them to understand how RFTS would be received and what modifications they may need to make to ensure success. Retailers highlighted that it was important to understand what customers wanted to buy, and for customers to feel supported so they could make a healthier choice. One retailer explained how RFTS helped make healthy food more visible in‐store:

If [customers] see it then they might want to buy it. Now, people came up and they want our help. (Retailer #1)

The importance of simplicity was a key sentiment expressed by both health promotion and retail staff members. Health promotion staff discussed the feedback they received from retailers after the initial pilot programme of RFTS. They explained that following this feedback, changes were made to how the in‐store materials were presented. Retailers had expressed frustration with some aspects of the programme, for example, they felt that maintaining shelf tags for individual products was too time‐consuming. Health promotion staff then adapted shelf tags to a single shelf strip label that streamlined the process for retail staff. Similarly, retail staff explained that they were usually very busy with the day‐to‐day running of their stores and didn't have time for additional tasks that were overly complicated. As a retailer explained the constraints on their time:

I'm a bit time poor here, because there's not a lot of staff… I don't have a lot of time to do it all … So yeah, I'm just a bit under the pump with everything. And then the [RFTS] stuff on top of it makes it even harder. (Retailer #2)

Promotional materials that were simpler (such as static posters) were described as correctly maintained by health promotion staff, and retailers talked about simple materials as being easier to use, suggesting that ease was central to their engagement with the intervention. Simplicity in messaging was also important from the perspective of community members. They explained that when considering the unique community needs of the Latrobe Valley, they needed to ensure that their messaging was clear, as one community member explained:

I just think it's a simple, hopefully foolproof, situation to lead and guide people to better nutrition. (Community member #3)

This was reflected in health promotion staff discussing how their key message, that all fruit and vegetables are five stars, was well understood and received by customers due to the purposely simple messaging used.

### 3.3 Trusting Relationships Enabled Implementation and Change

All participants emphasised that the relationships between retailers, Latrobe Community Health Service and Latrobe Health Assembly were central to the success of RFTS, both during the initial intervention and in supporting its ongoing sustainability. All participants also described the positive outcomes of these partnerships and how working together and using the unique skills and perspectives of each group made the programme stronger and more effective, as one staff member explained:

It's been a fantastic experience for us to be involved in, particularly as a partnership project. We've all brought different strengths to this project. (Health promotion staff #2)

Retailers often spoke about the support provided by Latrobe Community Health Service staff as central to the programme's success. Staff members were able to build a ‘trusting relationship’ with retailers through actions such as providing them with training, completing real time (using the Store Scout App [42]) assessments of the healthiness of the retail environment and its merchandising to measure their progress, and encouraging them to change displays where necessary.

Retailers described health promotion staff as knowledgeable, helpful and facilitators in their stores' efforts to meet the benchmarks set through assessment tools like the Store Scout App. Health promotion staff explained how they tried to ‘hear those voices of the supermarkets’ in their work and involve retailers in changes to the programme design. They also described how providing feedback to retailers helped build trust and enabled further changes.

One retailer expressed a desire to do more work to promote healthy eating in their store and saw the partnership between themselves and the health promotion team as critical to achieving that vision. As they explained:

No big supermarket, no brand [will] promote these things…They don't care which [product] is, 5 star, 1 star, 2 star. They don't care. [Latrobe Community Health Service], that community part, is really helpful because they are only focusing on that [HSR] ranking. So their focus is very much clear. (Retailer #3)

This desire for greater change was also commented on by health promotion staff, who explained that one supermarket had independently created end‐of‐aisle displays for four and five‐star items.

There were also additional healthy option nudge trials conducted at one supermarket at their request, supported by the health promotion staff. As one retailer explained, retailers also desired change because they could see the benefits:

You need to have some retailers who take action, who can come up with the plan. (Retailer #3)

The need for wide collaboration with new partners was highlighted by many participants. Partnering with local organisations such as food relief groups, greengrocers, schools and sporting clubs was suggested to increase the profile of RFTS. Retailers highlighted their desire for greater publicity for the programme, through possible future events at their stores or collaboration to organise prize draws to encourage the community to shop at participating supermarkets. Community members also thought there was potential for further collaboration between participating supermarkets and schools to provide fruit for children, to not only highlight RFTS but also to provide an opportunity for children to try new fruits they may not have had before. One community member explained a previously successful promotion:

We've done like some cross promotions … It was like colouring in bits of watermelon and bits of fruit and matching up fruit, and like that sort of thing. And they were going out to some of the schools with information packs like to go to the teachers just so they could start like talking about [RFTS]. (Community member #2)

Health promotion staff described the interest that RFTS had generated among other agencies across Victoria, and their desire to create a Community of Practice to coordinate efforts to improve healthy options within supermarket retail, and share resources, ideas and discuss challenges. As one staff member explained:

There's a real role for someone to play, whether that's [state health promotion agency] or the Department of Health, someone at a state level to do some coordination across the state. I think that would be incredible. (Health promotion staff #2)

Health promotion staff explained that they had been approached by many other local governments, local public health units and community health promotion funded agencies asking for advice on how to facilitate an intervention such as RFTS, including how to engage with supermarkets to ‘get them on board’ and promote the benefits of such a programme. Health promotion staff shared their learnings and experiences with others, explaining that there could be future potential to formally provide materials and training on implementing a programme like RFTS to other groups.

### 3.4 Scaling Up Required Organisational Commitment and Investment

All health promotion staff discussed the difficulties with achieving long‐term sustainability of RFTS and other similar projects. Health promotion staff expressed their enthusiasm and passion for RFTS, and their perception that the programme was making a difference in the community, but they were concerned about securing ongoing funding. This was explained by one staff member:

I don't think if there's any lack of ideas, it's who's going to fund it, and where it goes from here. (Health promotion staff #1)

Retailers expressed a desire to continue with RFTS despite the programme finishing, explaining that they felt it was a ‘good thing’ for the community's health. However, while this was received positively by health promotion staff, they also acknowledged that this would mean greater funding and staffing requirements, as one staff member commented:

We obviously have to provide a level of resourcing behind it to help continue to support the owners to deliver this project. As much as we can teach them and empower them there's still a level of resourcing that sits behind all of that. (Health promotion staff #2)

To mitigate this, health promotion staff explained how they were aiming to transition to a supermarket‐led model, where they would support rather than lead future work with these stores. Additionally, the discussion included using non‐traditional resources (such as university students on placements) for data collection and retailer engagement to reduce the required staffing resources.

Health promotion staff described that aligning supermarket store policies with health promotion objectives was important for sustaining the programme, explaining that endorsement from head office could support existing retailers and help recruit new ones. Retailers had explained that due to the supermarket corporate structure, they were unable to make changes to their individual store policy to reflect their participation in RFTS, instead aiming to change the store's practices. Health promotion staff explained that while this was challenging, they intended to invite the supermarket head office to future workshops to involve them in the programme and hopefully achieve head‐office level policy change to affect all supermarkets within the state. As a health promotion staff member explained:

[We are] aiming to produce an organisational food nutrition policy for the [independent supermarket association]. So outlining how they're going to continue to work towards a healthier food retail environment with some specific actions listed in that. (Health promotion staff #2)

Methods to scale up the programme and introduce it to new stores and areas were discussed by health promotion staff. They explained that a key enabler to greater programme reach would be to involve more supermarkets, including if possible major Australian supermarket chains such as Coles and Woolworths. Involving more supermarkets would allow a greater proportion of the Latrobe Valley community to engage with RFTS, as community members discussed that the involved supermarkets were not where they lived or bought groceries. Smaller supermarkets like those involved in RFTS were described by community members as not where most other community members did most of their grocery shopping, which reduced the overall exposure to RFTS promotional materials.

Community members described many ideas for future directions and expansions of the programme. One community member discussed considerations for the continuation of the programme:

Who should be involved moving forward? And how do we attract them moving forward? So you've got the supermarket owners. But we need to have a partner there that's going to be able to support them moving forward. (Community member #1)

Possible future partners that were discussed by the group included local sporting clubs, food relief agencies and corporate sponsors. The community members explained that while they felt the current programme was good, if it could go further and reach more people it could have greater impact. They suggested concepts such as ‘grab and go’ kits for customers, where they would have all the ingredients required for a fast, healthy meal, packaged and ready for them to purchase. Additionally, they discussed new ways of appealing to customers who may be unsure about healthy foods, by highlighting other benefits of healthy products such as their seasonality, that they were more economical or better value, and that they could prepare them in ways that used less electricity.

Similarly, health promotion staff also had ideas for new ways they could expand the existing RFTS programme to continue its growth ‘from strength to strength’. They discussed the importance of collecting sales data in the future, and how this could help to prove the effectiveness of RFTS from a business perspective by demonstrating that the programme improved or did not affect overall sales.

Health promotion staff explained that RFTS was one part of a greater advocacy effort they were leading for their community. They explained how their involvement in this programme had led to other advocacy efforts, such as creating submissions to government consultations regarding unhealthy food marketing to children, and the health star rating system. Health promotion staff described this work as the beginning of their advocacy efforts and that they had identified a need for greater support for their community, as a staff member commented:

We'll continue to look for opportunities to advocate, and also advocate for more healthy food retail initiatives like Reach for the Stars. (Health promotion staff #2)

## 4 Discussion

Our study interviewing 12 participants including community members, health promotion staff and retailers found that participants had overall positive experiences with the RFTS programme, particularly focusing on the importance of designing the intervention with their local community to ensure that their needs were considered and the value they placed on the relationships formed. Additionally, participants highlighted sustainment‐related challenges with securing long‐term funding for the programme to provide continued support for retailers from health promotion staff. Longer‐term planning and next steps for the programme included up‐scaling to other stores, other local government areas and continuing advocacy to improve healthy food access for their community.

Participants in our study highlighted the importance of community engagement, and the value of partnerships and collaborations in both designing and implementing RFTS. RFTS was initially co‐created through consultation with the Latrobe Valley community and so was able to be tailored specifically to the needs and priorities of the people who would be part of the programme. This collaborative approach has been shown to be successful with community‐based food retail programmes [43, 44]. Recent systematic reviews of methods used in co‐design have shown that using a co‐design approach can be useful to promote collaboration, and can enhance the development of public health interventions, tools or services [45, 46]. Co‐design has been used to increase the effectiveness of public health tools, interventions and services by including the intervention or programme population in the design and implementation [46]. Co‐design aims to identify and meet the unique needs of specific populations or communities, through empowering them to use their experiences and opinions to provide a sense of ownership over the programme, thereby creating trust and confidence [45]. Our study found evidence that using a co‐design approach to develop the RFTS programme resulted in strong support for the programme, with health promotion staff and community members explaining how they designed the programme and developed the messaging in a way that would be understood by their local community. RFTS was designed to be more accessible to various population groups, including those with lower education, reflecting the demographics of the area [32]. The programme also provided simple healthy recipe cards for shoppers to follow, after community consultation highlighted this as something they wanted. Additionally, involving retailers as part of the co‐design process ensured that RFTS was something they could integrate into their regular practice, without overburdening them with time‐consuming activities that they would not be able to follow or update. This ongoing retail engagement contrasts with other community‐organisation‐led strategies in supermarkets, where retailers' interest fades, limiting the chances to sustain health‐enabling strategies in‐store [47]. A systematic umbrella review summarised the factors influencing implementation and scalability of healthy food retail interventions highlighted the importance of including retailer perspectives [27]. However, other studies have highlighted how researchers and health promotion staff must find the balance between appealing to business interests, without letting them lead programme design, as this can compromise the health benefits of the intervention by prioritising commercial outcomes over community health needs [48].

Securing ongoing funding to sustain public health interventions and programmes is a common problem. A previous qualitative study interviewing public health professionals found that often when funding is discontinued, the programme's reach and capacity are diminished [49]. In Australia, it is difficult to secure long‐term funding for public health or health promotion programmes, as this funding is often vulnerable to shifts in political priorities and broader budgetary constraints, and there is overall low government investment in preventive health measures [50]. Health promotion has been described as a ‘soft target’ in times of fiscal pressure, with resources reallocated to address immediate health system demands, even when this may undermine longer‐term population health goals [51]. This is despite international evidence suggesting that community‐level public health interventions provide a strong return on investment and cost–benefit ratio [52]. Another common challenge in maintaining public health interventions can be when a key person (the ‘champion’) leaves the organisation, and interest and enthusiasm for the programme wane [53]. Health promotion staff tried to minimise this potential impact for RFTS by aiming to transition to a ‘supermarket led’ model, where primary responsibility for the programme would be held by the retailers. This would have the additional benefit of reducing health promotion staffing resources required to continue delivering RFTS. To help with ongoing sustainability health promotion staff also highlighted a desire to involve the head office for the independent supermarkets to transition RFTS from practice into an official part of store policy. A previous study using food retailer interviews showed that organisational‐level change, supported by leadership commitment and dedicated resourcing, can strengthen the long‐term sustainability of health‐related interventions and programmes [54]. Over time, these structural supports can create a reinforcing cycle in which demonstrated success at the store level encourages further organisational investment [54]. Recent efforts to consolidate lessons in this field, such as the development of a best practice guide for conducting healthy food retail research [55], also emphasise the importance of leadership commitment, retailer engagement and clear structural supports as central to sustaining interventions. Our findings reinforce and extend this guidance by providing qualitative insights into how these dynamics are experienced in practice.

### 4.1 Study Strengths and Limitations

This study's strength lies in the depth and nuance of insight it provides into how key interest‐holders experienced and understood the sustainability of a supermarket‐based health promotion programme. The use of a reflexive, constructionist approach facilitated detailed exploration of the relational, organisational and contextual dynamics at play, producing findings that illuminate the complexities of sustaining public health initiatives in retail settings. Additionally, this study examines RFTS after an extended period of implementation over 2 years. The sustained length of the intervention is relatively novel in supermarket‐based health promotion research and provided rich material for participants to reflect on, generating nuanced insights into factors that support or constrain programme sustainability.

However, our study does have some methodological limitations. As with all qualitative research, the findings of this study represent one situated and contextualised account, shaped by the perspectives of the participants and the interpretive lens of the researchers. This study sought perspectives from community members, retailers and health promotion staff involved in Reach for the Stars. While all interest‐holder groups were represented, the number of participants and format of data collection differed across groups, resulting in variation in the volume of data generated. Consequently, some themes were informed more strongly by particular interest‐holder groups than others. In addition, one retailer participant declined audio recording, requiring reliance on detailed interview notes rather than a verbatim transcript, which limited the availability of direct quotations from this participant. Additionally, a further limitation relates to the use of the voluntary Health Star Rating system, which has been criticised for selective uptake and occasional misalignment with broader dietary guidance. Ongoing public controversy about the government‐endorsed front‐of‐pack labelling system continues to pose challenges for promotional and labelling interventions. Finally, because the RFTS programme was co‐designed with the local community, some findings may be specific to this context, though other insights may be relevant to similar settings. The perspectives shared were influenced by participants' close involvement in RFTS and their commitment to its goals, which may have shaped how challenges and successes were discussed.

## 5 Conclusion

Sustaining supermarket‐based health promotion initiatives requires attention to relational, organisational and contextual factors beyond programme design alone. Participants in this study viewed trusting partnerships, active community involvement and straightforward, adaptable programme materials as enabling success, but also recognised that without secure funding and adequate staffing, maintaining and expanding these initiatives remains difficult.

## Author Contributions

R.B. was responsible for study conceptualisation, methodology, data collection, data analysis and drafting the manuscript. C.V. was responsible for study conceptualisation, supervision, methodology, data collection and reviewing and editing the manuscript. L.D., K.M., S.A., B.B., M.B. and S.N. were responsible for reviewing and editing the manuscript.

## Funding

Reach for the Stars (RFTS) is a partnership project between Latrobe Community Health Service and Latrobe Health Assembly. This work was funded as part of the Latrobe Health Innovation Zone Initiative funded by the Latrobe Health Assembly in partnership with the Victorian Government. The funder did not have any role in the analysis or interpretation of the data, preparation of the manuscript or the decision to submit the manuscript for publication. L.D. is supported by Latrobe Community Health Service. K.M. is supported by Latrobe Health Assembly. R.B., C.V., M.B., B.B., S.N., A.C. and S.A. are researchers within the National Health and Medical Research Council (NHMRC) Centre of Research Excellence in Food Retail Environments for Health: Next Generation (APP2024716). The opinions, analysis and conclusions in this paper are those of the authors and should not be attributed to the NHMRC.

## Ethics Statement

Ethics approval was obtained from Deakin University's Human Research Ethics Committee (2024/HE000289). All participants provided informed consent to participate. All but one participant consented to audio recording and the use of non‐identifiable quotes; for the interview conducted without recording, detailed notes were taken by RB with the participant's consent.

## Conflicts of Interest

The authors declare no conflicts of interest. This work was funded by Latrobe Health Assembly, however the funder did not have any role in the analysis or interpretation of the data, preparation of the manuscript or the decision to submit the manuscript for publication.

## Supporting information


**File S1:** Study interview guide.

**File S2:** Standards for reporting qualitative research checklist.

**File S3:** Complete list of codes used in the reflexive thematic analysis.

## Data Availability

The data that support the findings of this study are available on request from the corresponding author. The data are not publicly available due to privacy or ethical restrictions.
