# Supplementary material for: Sustaining Health Promotion in Australian Supermarkets: Qualitative Perspectives From the Reach for the Stars Programme
Source: Health Promot J Austr. 2026 Aug 11;37(4):e70228. doi: 10.1002/hpja.70228 (PMC13459042; doi:10.1002/hpja.70228)
Supplement: Supplementary file 2 [file HPJA-37-0-s002.docx]

Sustaining Health Promotion in Australian Supermarkets: Qualitative Perspectives from the Reach for the Stars Program

Supplementary File 3 – Codebook

| Name | Description | Files | References |
| --- | --- | --- | --- |
| Challenges - health promotion staff |  | 1 | 5 |
| **Change in customer behaviour** |  | 1 | 1 |
| **Community of Practice** |  | 2 | 3 |
| **Continue relationships with new health promotion staff members** |  | 1 | 1 |
| **Customer choose what they want** |  | 1 | 2 |
| **Design was community led** |  | 2 | 2 |
| Difficulties |  | 1 | 2 |
| **Education is difficult** |  | 4 | 8 |
| **Future growth, new community partners** |  | 2 | 8 |
| Health promotion hope retailers see value in program |  | 2 | 2 |
| **History of project** |  | 1 | 2 |
| **How health promotion supports** |  | 2 | 12 |
| **How to scale up** |  | 2 | 13 |
| **Ideas for the future** |  | 1 | 13 |
| **Imbed intervention into usual practice** |  | 2 | 2 |
| **Impact on community** |  | 2 | 3 |
| **Importance of codesign with reatilers and community** |  | 1 | 3 |
| **Importance of knowing your customers** |  | 2 | 2 |
| **Importance of partnerships** |  | 1 | 1 |
| **Importance support of health promotion staff to retailers** |  | 4 | 9 |
| **Initial difficulties** |  | 1 | 4 |
| **Involve supermarkets in design** |  | 1 | 2 |
| Mixed customer feedback |  | 1 | 1 |
| **More publicity** |  | 3 | 4 |
| **Motivation to continue the program** |  | 2 | 2 |
| **Needed for Latrobe valley** |  | 5 | 14 |
| New Code |  | 0 | 0 |
| **New codesign process to change store policy** |  | 1 | 3 |
| **New data collection, sales data** |  | 1 | 1 |
| No feedback from customers |  | 2 | 2 |
| **No impact on store policies** |  | 4 | 5 |
| **Oppourtunities for advocacy** |  | 2 | 6 |
| **Positive experiences - health promotion staff** |  | 1 | 3 |
| **Positive experiences - retailers** |  | 4 | 6 |
| **Possibly losing impact over time** |  | 1 | 2 |
| **Postiive customer feedback** |  | 1 | 2 |
| **Problems with sales data collection** |  | 1 | 3 |
| **Progress motivates retailers** |  | 1 | 1 |
| Quotes |  | 4 | 13 |
| **Retailer related challenges** |  | 3 | 7 |
| **Retailer wanting to do more** |  | 2 | 4 |
| **Retailers maintaining intervention materials** |  | 2 | 2 |
| **Sharing resources** |  | 1 | 1 |
| **Simple measures most effective** |  | 5 | 12 |
| Small impact |  | 1 | 1 |
| **Supermarket-led transition** |  | 2 | 2 |
| **Supermarkets want to help** |  | 1 | 1 |
| **Support needed from IGA head office** |  | 1 | 4 |
| **Sustainability challenges** |  | 2 | 10 |
| **Tailored to the community** |  | 2 | 4 |
| **Teaches customers about healthy eating and health star rating** |  | 3 | 4 |
| **Types of data for future projects** |  | 1 | 1 |
