# Supplementary material for: Sustaining Health Promotion in Australian Supermarkets: Qualitative Perspectives From the Reach for the Stars Programme
Source: Health Promot J Austr. 2026 Aug 11;37(4):e70228. doi: 10.1002/hpja.70228 (PMC13459042; doi:10.1002/hpja.70228)
Supplement: Supplementary file 3 [file HPJA-37-0-s003.docx]

## Sustaining Health Promotion in Australian Supermarkets: Qualitative Perspectives from the Reach for the Stars Program

## Supplementary File 1

## Interview guide – Reach for the Stars

1. Self-introduction
2. Plain language explanation of the interview, objectives, dynamic of the session, and length of the session.
3. Verbal confirmation of consent
4. Collection of consent forms
5. Participant introduction

| Introduction | Can you tell me a bit about your role? |
| --- | --- |
|  | Can you talk to me about the Reach for the Starts program?  *Prompts: how did it all get started?* |
|  | Do you think a program like RFTS was needed in the Latrobe Valley?  *Why? Why this area?* |
|  | What was the process for preparing for Reach for the Stars in your store?  *For Health Promotion officers: how did you help to prepare retailers to participate in RFTS?* |
|  | What was your first impression when RFTS was introduced?  *For retail owners: what were your staff’s initial impression?* |
| Reflections and perceptions of RFTS | How did you find participating in the program, overall? |
|  | Which part of the program (shelf labelling, the prominent displays, signage, etc) do you think was the most effective?  *Was there one element that you think worked especially well? Why?* |
|  | Did you notice any changes in customer behaviour or purchasing since RFTS began?  *For health promotion officers: did you get feedback from retail owners about changes in customer purchasing since RFTS began?* |
|  | What did you find difficult?  *What did you find helpful to make the required changes?* |
|  | If you could change one thing about this program, what would it be?  *Why?* |
|  | Did you receive any feedback from customers that could help the program to improve?  *What was it?* |
|  | How do you think this program is different from other promotions/campaigns/etc  *Do you think that makes it more/less successful?* |
|  | Did RFTS have any impact on your in-store policy? Why?  *For health promotion officers: do you think RFTS had any impact on in-store policy, why?* |
| Sustainability of RFTS | Can you explain how you feel to continue to promote healthier items in your store, even beyond this program? Why is that? |
|  | When RFTS ends, can you talk to me about what would help you to continue to promote healthier choices in your store independently? *For health promotion officers: what kind of supports do you think retailers need to maintain healthy changes?* |
|  | Do you think this program could be financially sustainable without outside funding or support?  *If so, how? If not, what would be needed?* |
|  | Can you think of any other partnerships, initiatives or community programs that could help to sustain this in the long term? |
|  | Do you think there are any other people we should involve in this type of initiative?  *Do you think that they would help to sustain the program?* |
|  | Are there any ways we could modify this program to better suit specific health needs in the community in the Latrobe Valley? |
| Closing | What lessons do you think can be learned from RFTS? |
|  | From your perspective, can you talk to me about how programs like this might have the potential to impact the community’s health? |
|  | Do you have any advice for us about how we could scale up this program to other communities in Victoria? |
|  | In research, we talk about “co-creating” programs and initiatives by involving people like you from the very beginning of our planning of new initiatives. Do you think that using this approach in the future might be helpful/make the initiative better?  *Why?* |
|  | Before we wrap up, was there anything else that you would like to add? |

**Closing**

6. Summary of the session
7. Commitment to sending the final diagram for review and feedback

**Reflection questions for the interviewer**

1. In general, how did the session go?
2. Did it proceed as expected? Why?
3. What is the most memorable part of the session?
4. What do you think was the most relevant question for the participant? Why?
